# Evolutionary relationship of fat body endoreduplication and queen fecundity in termites

**DOI:** 10.1002/ece3.5664

**Published:** 2019-09-27

**Authors:** Tomonari Nozaki, Kenji Matsuura

**Affiliations:** ^1^ Laboratory of Insect Ecology Graduate School of Agriculture Kyoto University Kyoto Japan; ^2^ Center for the Development of New Model Organisms National Institute for Basic Biology Okazaki Japan

**Keywords:** caste differentiation, flow cytometry, Isoptera, ploidy variation, queen physogastry, social insects

## Abstract

Endoreduplication or nuclear genome replication without cell division is widely observed in the metabolically active tissues of plants and animals. The fat body cells of adult female insects produce abundant yolk proteins and become polyploid, which is assumed to accelerate egg production. Recently, it was reported that in termites, endopolyploidy in the fat body occurs only in queens but not in the other females; however, the relationship between the fecundity and ploidy level in the fat body remains unclear. Termite queens exhibit a huge variation in their egg‐producing capacity among different species; queens in the species with a foraging lifestyle, in which workers leave the nest to forage outside, are much more fecund than those in the species living in a single piece of wood. In this study, we conducted ploidy analyses on three foragings and three wood‐dwelling termites via flow cytometry. In all the species, the fat body of queens contained significantly more polyploid cells than that of other nonreproductive females, considering their body size effect. However, the male fat body, which is not involved in yolk production, did not show consistency in polyploid cell numbers among the species studied. Moreover, highly fecund queens in foraging termites exhibit higher levels of endopolyploidy in their fat body than those with less fecundity in wood‐dwelling termites. These results suggest that endopolyploidy in the fat body of termite queens can boost their egg production, and the level of endopolyploidy in their fat body is linked to their fecundity. Our study provides a novel insight into the evolutionary relationship between endoreduplication and caste specialization in social insects.

## Graphical abstract

Our study links a tissue‐specific ploidy elevation to reproductive division of labor in social insects; ploidy analysis via flow cytometry across six phylogenetically diverse termites revealed that the fat body of queens contained more polyploid cells than that of other nonreproductive females. Furthermore, highly fecund queens in foraging termites exhibit higher levels of endopolyploidy in their fat body than those with less fecundity in wood‐dwelling termites. Fat body produces massive amount of yolk proteins; thus, our results suggest that the endoreduplication is regulated in accordance with the metabolic demand in termites.

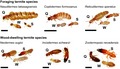

## 1 INTRODUCTION

Social insects are defined by the reproductive division of labor, wherein a small number of individuals monopolize reproduction while others conduct nonreproductive tasks such as foraging, brood care, defense, and maintenance of the colony (Wilson, 1971). Extreme reproductive specializations are exhibited in social insects under such a system. Female fertility of social insects ranges from complete sterility to hyperfecundity (Wheeler, 1996). For instance, exceptionally high fecundity was observed in queens of army ants and mound‐building termites (Schneirla, 1971; Wheeler, 1996; Wyss‐Huber & Lüscher, 1975), whereas the workers had greatly reduced reproductive potential. Morphological and physiological differences between reproductive and nonreproductive individuals, which are generated from the same genome, are prime examples of polyphenism (Wheeler, 1986) and have been attracting considerable attention for a long time (Costa‐Leonardo, Laranjo, Janei, & Haifig, 2013; Evans & Wheeler, 2001; Jemielity, Chapuisat, Parker, & Keller, 2005; Roma, Bueno, & Camargo‐Mathias, 2010; Weiner & Toth, 2012).

Endopolyploidy or somatic polyploidy is generated through endoreduplication cycles, in which the nuclear genome is replicated without subsequent cell division (Edgar & Orr‐Weaver, 2001; Edgar, Zielke, & Gutierrez, 2014; Lee, Davidson, & Duronio, 2010; Shu, Row, & Deng, 2018) and polyploid cells are typically observed in the tissues with high metabolic demand or rapid growth in most plants and animals (reviewed in Lee et al., 2010; Neiman, Beaton, Hessen, Jeyasingh, & Weider, 2015). Endoreduplication is often attributed to increasing cellular size, metabolism, and gene expression owing to the increasing availability of DNA templates for transcription (Bourdon et al., 2012; Claycomb, Benasutti, Bosco, Fenger, & Orr‐Weaver, 2004; Edgar et al., 2014; Galitski, Saldanha, Styles, Lander, & Fink, 1999; Nagl, 1976). In social hymenopterans, several studies have shown the intriguing linkage between caste specialization and endopolyploidy (Rangel, Strauss, Seedorf, Hjelmen, & Johnston, 2015; Scholes, Suarez, & Paige, 2013; Scholes, Suarez, Smith, Johnston, & Paige, 2014). Scholes et al. (2013) investigated the degree of polyploidy within and among castes of four ant species with worker polymorphism and observed variation in ploidy levels among workers with different body sizes. Rangel et al. (2015) surveyed transition of polyploid levels in several body parts of worker honey bees and found age‐related tissue‐specific changes in endopolyploidy levels in the honey bees of different ages. However, there are very few reports on the relationship between endopolyploidy and the division of labor in reproduction.

Recently, we observed that the queens of the termite, *Reticulitermes speratus* Kolbe, exhibited higher endopolyploidy levels in their fat body cells in comparison with the nonreproductive females (Nozaki & Matsuura, 2016). Insect fat body is a multifunctional organ, which combines the roles of many tissues including the liver and fat tissues in vertebrates. This tissue is involved in the synthesis, storage, and secretion of lipids, proteins, and carbohydrates (Arrese & Soulages, 2010). During oogenesis, the female fat body produces abundant yolk protein precursors, namely vitellogenins (Bownes, 1986; Tufail & Takeda, 2008). The rate of vitellogenin synthesis from internal nutrients is probably one of the essential factors determining the capability of egg production. It has long been known that in certain solitary insects, the fat body cells of sexually mature females become polyploid, presumably to promote vitellogenin synthesis (Dittmann, Kogen, & Hagedorn, 1989; Irvine & Brasch, 1981; Nair, Chen, & Wyatt, 1981). These studies suggest that the fat body endopolyploidy enhances the reproductive specialization of queens for egg production; however, other possibilities remain to be explored. For example, not vitellogenin synthesis, but simply body size differences among individuals can also affect the ploidy level in the fat body (Scholes et al., 2013). The ploidy levels of the male fat body, which is not involved in vitellogenin synthesis, should also be examined (Terrapon et al., 2014). Determining the ploidy levels in fat body cells of both the sexes of various termite species would provide further implications for the functional significance of endopolyploidy in caste specialization and queen fecundity in social insects.

**Figure 1:**
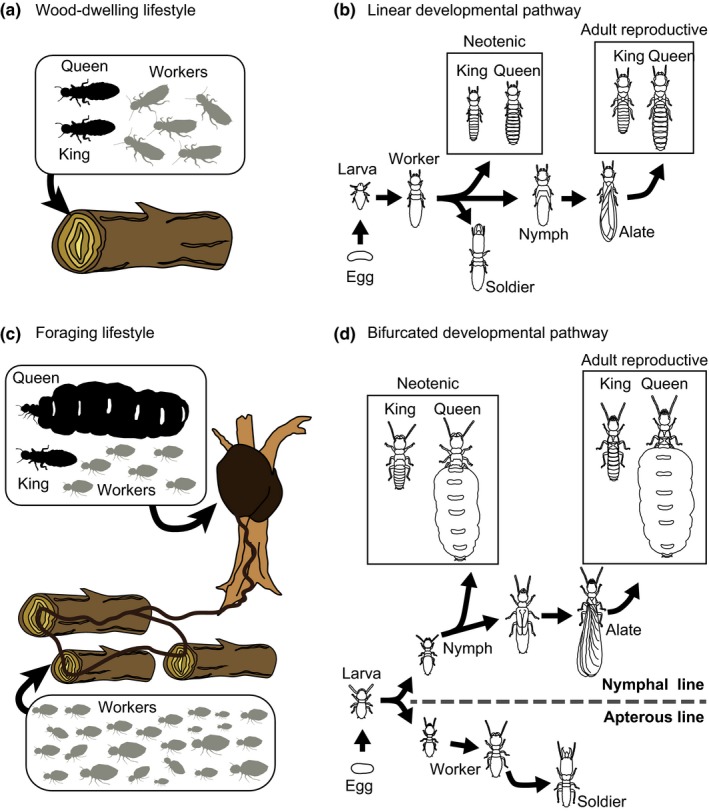
Life types of termites and developmental patterns. Wood‐dwelling species nest in a single piece of wood that serves both as a shelter and food source (a) and their postembryonic development was linear (b). Foraging species are characterized by worker foraging outside of their nest and resultant multiple‐pieces nesting (c). They display a bifurcated development between worker and reproductives (d)

In termites, queen fecundity varies greatly between lineages with different lifestyles. Wood‐dwelling termites utilize a single piece of wood, both as food source and shelter (Figure 1a; Korb & Hartfelder, 2008; Korb et al., 2015), and form small colonies from several hundreds to a few thousands (reviewed in Nutting, 1969). Wood‐dwelling lifestyle is considered phylogenetically basal (Korb et al., 2015), and a characteristic of damp‐wood termites (Archotermopsidae), dry wood termites (Kalotermitidae), and the ancestral clade of subterranean termites (Rhinotermitidae). In these species, both queens and workers share the same developmental pathway, that is, workers are actually immature and all of them can potentially develop into reproductive individuals (Figure 1b; Korb & Hartfelder, 2008). On the other hand, the foraging termites are characterized by multiple‐piece nesting (Figure 1c) and large colony size of at most several millions (reviewed in Nutting, 1969; estimated in Evans, Lenz, & Gleeson, 1998; Evans, Lenz, & Gleeson, 1999). The workers leave the nest to forage outside for food materials at some point after colony formation (Korb et al., 2015). This lifestyle is observed in the advanced clade of Rhinotermitidae such as *Coptotermes*, *Heterotermes*, and *Reticulitermes* (Bourguignon et al., 2015), and in all of the higher termites (Termitidae). These species exhibit an early developmental bifurcation between the worker and reproductive termites (Figure 1d; Korb & Hartfelder, 2008), which might enhance the physiological discrepancy between them. Queens of foraging species exhibit much higher fecundity than those of the wood‐dwelling species (reviewed in Nutting, 1969; Weesner, 1969) and can unfold to expand their abdominal epicuticle of intersegmental membrane along with ovarian development (Myles, 1999). In the higher termites, it has been demonstrated that the abdomen of fully matured queens would be distended five to eight times its original size and their intersegmental membrane not only unfold but also grow continuously (Bordereau, 1982; Bordereau & Andersen, 1978). These variations in queen fecundity among different termite species are likely a reflection of the factors surrounding a particular lineage such as their lifestyle. For example, termite queens with high fecundity could be more favorable in foraging species, but probably not as much in wood‐dwelling termites (Myles, 1999).

**Figure 2:**
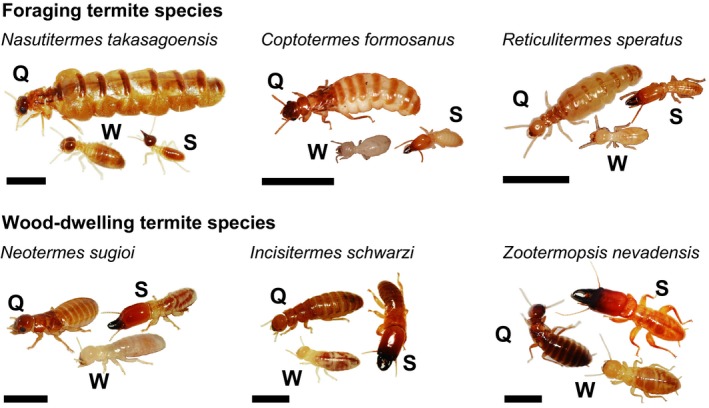
Photographs of sample species used in this study for nuclear DNA content analysis. Scale bars indicate 5 mm. Q, queen; S, soldier; W, worker

In this study, we investigated the relationship between reproduction and fat body polyploidization using three foraging species with highly fecund queens (Termitidae: *Nasutitermes takasagoensis* Shiraki; Rhinotermitidae: *Coptotermes formosanus* Shiraki and *Reticulitermes speratus* Kolbe; Figure 2a) and three wood‐dwelling species with less fecund queens (Kalotermitidae: and *Neotermes sugioi* Yashiro and *Incisitermes schwarzi* Banks; Termopsidae: *Zootermopsis nevadensis* Hagen; Figure 2b). These species roughly cover the termite phylogenetic diversity and exhibit considerable variations in the body mass of queens (Table 1). First, we investigated the effect of caste (especially, queens vs. nonreproductive females) and body weight on the polyploid level in the fat body for each species. We also analyzed the endopolyploidy levels in the male fat body that is not involved in vitellogenin synthesis (Terrapon et al., 2014). Then, in order to elucidate the relationship between endopolyploidy and queen fecundity, we compared the relative ploidy levels of queens with the ploidy levels of conspecific workers between queens of the foraging and the wood‐dwelling species.

**Table 1:** Body weight distribution of sample individuals used for ploidy analysis

Species	Body weight distribution (mg)				
	Queen	King	Worker	Soldier	Others
*Nasutitermes takasagoensis*	84.9–147.1 (5)	11.0–14.9 (5)	♀: 3.8–6.0 (5) ♂: 2.1–2.8 (5)	♂: 2.0–2.5 (5)	Nymph ♀: 12.5–15.1 (5) Nymph ♂: 10.8–12.9 (5)
*Coptotermes formosanus*	25.4–42.3 (5)	9.8–11.2 (5)	♀: 3.1–4.0 (5) ♂: 2.5–3.8 (5)	♀: 3.4–4.7 (5) ♂: 2.5–3.9 (5)	NA
*Reticulitermes speratus*	3.8–9.7 (5)	3.1–4.6 (5)	♀: 1.7–2.2 (5) ♂: 1.8–2.2 (5)	♀: 1.9–2.8 (5) ♂: 1.5–2.5 (5)	NA
*Neotermes sugioi*	17.6–30.4 (5)	16.7–22.2 (3)	♀: 10.5–20.3 (5) ♂: 11.9–20.6 (3)	♀: 17.2–23.5 (5) ♂: 11.9–20.6 (3)	NA
*Incisitermes schwarzi*	11.3–25.9 (5)	11.9–17.8 (5)	♀: 8.6–16.0 (5) ♂: 7.5–20.8 (5)	♀: 7.8–23.2 (5) ♂: 8.6–25.2 (5)	Alate ♀: 9.9–12.9 (3) Alate ♂: 8.0–11.4 (3)
*Zootermopsis nevadensis*	35.0–54.5 (5)	39.7–45.9 (5)	♀: 23.0–50.2 (5) ♂: 42.5–50.4 (5)	♀: 67.2–95.0 (5) ♂: 63.6–88.8 (5)	NA

Note

In this table, the number in parentheses shows the sample size and “Nymph” indicates the last instar nymph.

## 2 MATERIALS AND METHODS

### 2.1 Study species of termites

#### 2.1.1 *Nasutitermes takasagoensis* Shiraki (Termitidae; wood‐feeding higher termite, foraging species)

Seven mature colonies of *Na. takasagoensis* (A–G) were collected in the end of January 2016, from Iriomote Island, Okinawa Prefecture, Japan. This species is known to produce eggs even in the winter season (Matsuura & Yashiro, 2010). The carton nests were carefully transported to the laboratory and dissected, and termites were extracted. Termites were used for ploidy analysis immediately after extraction from the carton nest. Colonies A–E contained primary royal pairs (both king and queen); however, F and G did not. The last instar nymphs were involved in colonies A–C, and F and G, whereas not in colonies D and E. All collected royals were adult (alate‐derived) reproductives, as previously reported (Hojo, Koshikawa, Matsumoto, & Miura, 2004).

#### 2.1.2 *Coptotermes formosanus* Shiraki (Rhinotermitidae; subterranean termite, foraging species)

In June 2013 and 2014, two *C. formosanus* colonies comprising newly emerged alates were collected from a coastal pine forest in Gobo City, Wakayama Prefecture, Japan and brought to our laboratory. The colonies were maintained in plastic containers at 25°C until the alates emerged and flew. After swarming, the alates were separated based on their sex, identified by the morphology of caudal sternite (Zimet & Stuart, 1982). Thereafter, they were placed into Petri dishes (ca. 90 × 20 mm) with a moist, unwoven cloth. To obtain the reproductives of this species, new colonies were established and reared as follows. Female–male pairs were haphazardly chosen from the same natal colony, and each pair was placed in a plastic Petri dish (ca. 55 × 20 mm) containing mixed sawdust bait blocks (Matsuura & Nishida, 2001; Nozaki, Yashiro, & Matsuura, 2018), which was kept at 25°C in constant darkness. Approximately 3 years later, each incipient colony was transferred into a plastic box containing additional wood chips and commercial culture soil (Tsuchitarou, Sumirin Nousan, Japan). In September 2018, five of the colonies were used for the ploidy analysis. All these five colonies comprised adult royals, and the queens' abdomens were expanded, and ovaries were fully developed. The number of individuals including workers and soldiers was more than 1,000 (from 1,033 to 3,147), and numerous egg piles were present in their nests; hence, we treated the royals from these colonies as fully matured reproductives.

#### 2.1.3 *Reticulitermes speratus* Kolbe (Rhinotermitidae; subterranean termite, foraging species)

From June to August 2018, five *R. speratus* colonies were collected from pine and cedar forests of Kyoto and Shiga Prefecture, Japan. In *R. speratus*, egg production is seasonal (Matsuura, Kobayashi, & Yashiro, 2007). We used colonies collected during the egg‐producing season. Rotten woods containing their colonies were transported to the laboratory, and the extracted termites were immediately used for ploidy analysis. Each colony contained multiple queens, which were nymph‐derived neotenics, and one adult king, as reported in this species (Matsuura et al., 2018).

#### 2.1.4 *Neotermes sugioi* Yashiro (Kalotermitidae; drywood termite, wood‐dwelling species)

Two colonies of *Ne. sugioi* were collected in February 2016 (A and B) and four in January 2017 (C–F) from mixed forest and a public park in Uruma City and Kunigami District, Okinawa Prefecture, Japan. Dead branches containing termites were carefully transported to the laboratory. Extracted termites were placed into Petri dishes (ca. 90 × 20 mm) with a moist, unwoven cloth and used for ploidy analysis within 3 days after extraction. Colonies C and D comprised royal pairs; colonies A, B, and E contained only queens, and colony F had only kings. All collected royals were adults, that is, alate‐reproductive termites. Queens’ ovaries were fully developed, and they contained mature oocytes in each ovariole.

#### 2.1.5 *Incisitermes schwarzi* Banks (Kalotermitidae: drywood termite, wood‐dwelling species)

Five colonies of *I. schwarzi* were collected in August 2015 (A–C) and 2016 (D and E) from Minamidaito Island, Okinawa Prefecture, Japan (introduced population, see Ide, Kanzaki, Ohmura, Takematsu, & Okabe, 2016). Dead branches and dry dead woods containing their colonies were carefully transported to the laboratory. All termites were extracted and used immediately for ploidy analysis. All colonies comprised one pair of royals; however, here were both adult and neotenic reproductives. In detail, neotenic royal pairs were present in colonies A and B, adult royal pairs in colonies D and E, and adult queen and neotenic king in colony C. During extraction of termites, eggs were observed in all colonies and all queens had mature oocytes in their ovaries. Both types of queens can be functional in the species, therefore, we treated them as “queens.”

#### 2.1.6 *Zootermopsis nevadensis* Hagen (Archotermopsidae: damp‐wood termite, wood‐dwelling species)

Five *Z. nevadensis* colonies were collected in September 2015, from the mixed forest in Kawanishi City, Hyogo Prefecture and Ikeda City, Osaka Prefecture, Japan, where this species was introduced from North America (Yashiro, Mitaka, Nozaki, & Matsuura, 2018). Rotten woods containing their colonies were transported to the laboratory. Further, termites were extracted and used immediately for ploidy analysis. All used colonies contained one pair of royals, and the both king and queens were adult reproductives. During extraction of termites, numerous eggs were observed in all colonies and all queens had mature oocytes in their ovaries.

For all the six species, we used only one haphazardly selected individual belonging to each caste from each colony. Five individuals belonging to each caste were used for flow cytometric analysis, except for alates of *I. schwartzi* and kings, male workers, male soldiers of *Ne. sugioi* (only three colonies were available in both species, Table 1). In case of *Ne. sugioi*, we used only males from the colony without queens, and only females from the colony without kings. In colonies F and G of *Na. takasagoensis*, only last instar nymphs were used for analysis. As aforementioned, because colonies of *R. speratus* contain multiple queens, we chose one queen haphazardly from each colony.

All sampled individuals were sexed based on their caudal sternite configuration (Zimet & Stuart, 1982) except for *Na. takasagoensis*. In this species, the sex of late instar nymphs and alates can be determined based on the shape of their abdominal sternites, whereas such a method is not applicable to the workers. In this study, we followed Hojo et al. (2004) and Miura, Roisin, and Matsumoto (1998), wherein major workers classified as females and minor workers as males. The soldiers were always differentiated from the male workers in mature colonies; thus, only male soldiers were available in this species (Hojo et al., 2004; Toga, Minakuchi, & Maekawa, 2017).

### 2.2 Ploidy analysis

**Figure 3:**
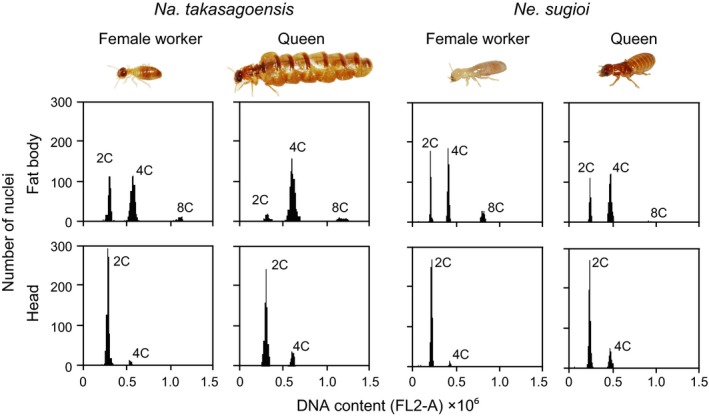
Examples of nuclear DNA content analysis by flow cytometry. The first peak corresponds to the distribution of 2C‐DNA nuclei, whereas the second and the third correspond to the distribution of 4C‐ and 8C‐DNA nuclei, respectively. The ploidy was determined by analysis of sperm cells (haploid; 1C) of conspecific kings

Fresh body weight of individual termites was measured with a digital balance in milligrams, to 2 decimals. The fat bodies of both sexes of reproductives (kings and queens), workers, and soldiers were dissected in phosphate‐buffered saline with fine forceps under a stereomicroscope (Olympus SZX7, Olympus). Similarly, we dissected last instar nymphs of *Na. takasagoensis* and alates of *I. schwartzi*. Typically, the fat body of termites appeared as whitish loose tissue located around the digestive tube and reproductive organs (Costa‐Leonardo et al., 2013). With utmost care and attention to avoid contamination by other tissues such as Malpighian tubules and tracheoles, we dissected the fat body from the abdomen of insects. In addition, we used the heads of all analyzed individuals as diploid tissue (Nozaki & Matsuura, 2016). Tissue for flow cytometric analysis was processed with a Cycletest PLUS DNA Reagent Kit (Becton Dickinson). All procedures were performed according to Nozaki and Matsuura (2016). Stained nuclei were analyzed for DNA‐PI fluorescence using an Accuri C6 Flow Cytometer (Becton Dickinson) at an excitation wavelength of 488 nm. Approximately, 1,000 cells were acquired for each measurement. Gating was performed using Accuri C6 software v1.0.264.21 to measure the nuclei count at each ploidy level (2C, 4C, and 8C) per sample; see Figure 3 for sample histograms. Based on the analyses of king's testis (sperm), we determined the 1C‐DNA peak, followed by 2C, 4C, and 8C peaks.

### 2.3 Statistical analysis

To determine whether the endopolyploidy level correlated with the castes, the proportion of polyploid cells in the fat body, which was calculated by the nuclei count with *C*‐value of 2C as diploid, and 4C, 8C and higher as polyploid, were compared among different castes within the species. Proportions of polyploid cells of each caste were analyzed using generalized linear mixed‐effect models (GLMM) with binomial errors and a logit‐link function, followed by Tukey's HSD post hoc test. Simultaneously, to examine the effect of body size on endopolyploidy, we included the body weight of each individual into the model. In this analysis, the castes and body weight were treated as a fixed effect and the original colony was included as a random effect. To avoid problems of nonconvergence, we included the optimizer “bobyqa.” We analyzed the males and females separately, because there must be functional differences between the sexes. Sex‐specific difference in gene expression pattern in the fat body has in fact been described previously (Mitaka et al., 2018).

To reveal whether the endopolyploidy level of queens differed among the species, especially between queens of foraging species and those of wood‐dwelling species, we examined the effect of lifestyles (foraging and wood‐dwelling) and the difference between the body weight of queen and female workers on the relative polyploid levels of queens. The relative polyploid level for each colony was calculated as follows:

$$\text{Relative}\;\text{polyploid}\;\text{level}=\log\frac{P_{q}\times \left(1-P_{w}\right)}{P_{w}\times \left(1-P_{q}\right)}$$

in which *P*_q_ is the proportion of polyploid cells in the fat body of queens and *P*_w_ is the proportion of polyploid cells in the fat body of female workers. The relative polyploid level of queens is the standardized polyploidy level of queens relative to the polyploid levels of conspecific female workers that represent nonreproductive females. This index is identical to the log odds ratio (logit) of the proportion of polyploid cells in queens versus workers, therefore, a normal distribution can be assumed for this index. The relative polyploid levels of queens were analyzed by linear mixed‐effect models (LMM), in which the lifestyles and body weight differences were handled as a fixed effect and species was included as a random effect. All analyses were conducted using the “car” (Fox & Weisberg, 2011), “lme4” (Bates, Maechler, Bolker, & Walker, 2015), and “multcomp” (Hothorn, Bretz, & Westfall, 2008) packages in R v3.5.1 (R Core Team, 2018).

## 3 RESULTS

### 3.1 Comparison of the proportion of polyploid cells among castes within species

**Figure 4:**
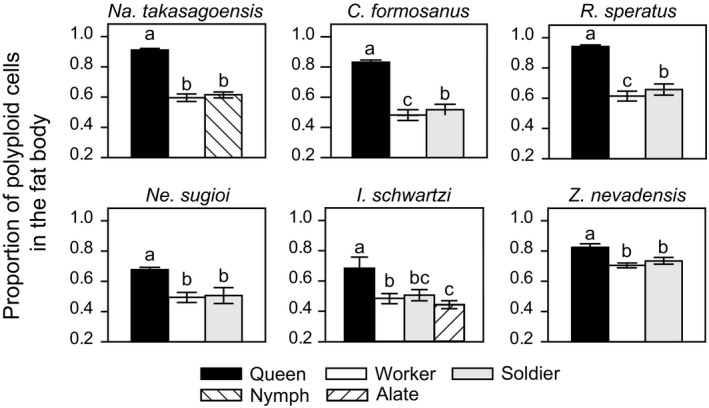
The proportion of polyploid cells in the fat body among females. Error bars represent the standard error of mean. Different letters indicate significant differences among means in each comparison group (Tukey's HSD test, *p* < .05)

Our flow cytometric analysis of female termites revealed that in all the six species studied, the queen fat body comprised a higher number of polyploid cells (4C and 8C) than diploid cells (2C), whereas that of other nonreproductive females contained roughly the same number of diploid and polyploid cells (Figures 3 and 4). Notably, only in *R. speratus*, the queen fat body contained a few cells with 16C‐DNA. In *Na. takasagoensis*, both castes and body weight had significant effects on the proportion of polyploid cells in the fat body (GLMM with a Wald chi‐square test, caste: *χ* ^2^ = 27.021, *df* = 2, *p* < .001, body weight: *χ* ^2^ = 4.433, *df* = 1, *p* = .035). In *C. formosanus*, whereas the effect of castes was significant (GLMM with a Wald chi‐square test, *χ* ^2^ = 71.8927, *df* = 2, *p* < .001), that of body weight was not significant (*χ* ^2^ = 2.7059, *df* = 1, *p* = .100). In *R. speratus*, the proportion of polyploid cells significantly differed among the castes (GLMM with a Wald chi‐square test, *χ* ^2^ = 197.800, *df* = 2, *p* < .001), yet body weight did not have any significant effect (*χ* ^2^ = 0.714, *df* = 1, *p* = .398). In *Ne. sugioi*, both castes and body weight had significant effects on the polyploid ratio in the fat body of female individuals (GLMM with a Wald chi‐square test, caste: *χ* ^2^ = 27.674, *df* = 2, *p* < .001, body weight: *χ* ^2^ = 23.508, *df* = 1, *p* < .001). In *I. schwartzi*, we found significant effects of both castes and body weight on the proportions of polyploid cells in the fat body (GLMM with a Wald chi‐square test, castes: *χ* ^2^ = 263.827, *df* = 3, *p* < .001, body weight: *χ* ^2^ = 60.772, *df* = 1, *p* < .001). In *Z. nevadensis*, while body weight had a significant effect on the proportion of polyploid cells in their fat body (GLMM with a Wald chi‐square test, *χ* ^2^ = 15.551, *df* = 1, *p* < .001), castes also had a significant effect (*χ* ^2^ = 216.074, *df* = 2, *p* < .001). In all species examined, queens presented significantly higher proportions of polyploid cells than that of other nonreproductives (Tukey's HSD, *p* < .05, Figure 4).

**Figure 5:**
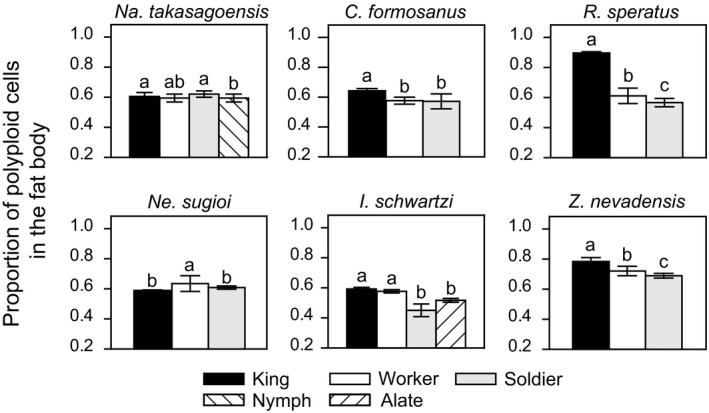
The proportion of polyploid cells in the fat body among males. Error bars represent the standard error of mean. Different letters indicate significant differences among means in each comparison group (Tukey's HSD test, *p* < .05)

On the other hand, in males, we did not find a consistent relationship between the caste and endopolyploidy in the fat body as observed in females (Figure 5). In *Na. takasagoensis*, we found significant effect of castes on the proportion of polyploid cells in the fat body of males (GLMM with a Wald chi‐square test, *χ* ^2^ = 24.753, *df* = 3, *p* < .001), yet body weight did not have significant effect on polyploidy (*χ* ^2^ = 1.818, *df* = 1, *p* = .178). In *C. formosanus*, both castes and body weight had significant effects on the proportion of polyploid cells in the fat body (GLMM with a Wald chi‐square test, caste: *χ* ^2^ = 10.953, *df* = 2, *p* = .004, body weight: *χ* ^2^ = 12.385, *df* = 1, *p* < .001). In *R. speratus*, while the effect of castes was significant (GLMM with a Wald chi‐square test, *χ* ^2^ = 213.7594, *df* = 2, *p* < .001), that of body weight was marginally nonsignificant (*χ* ^2^ = 3.812, *df* = 1, *p* = .051). In *Ne. sugioi*, we found significant effects of both castes and body weight on the proportions of polyploid cells (GLMM with a Wald chi‐square test, castes: *χ* ^2^ = 37.630, *df* = 2, *p* < .001, body weight: *χ* ^2^ = 35.304, *df* = 1, *p* < .001). In *I. schwartzi* males, both castes and body weight had significant effects on the proportion of polyploid cells in the fat body (GLMM with a Wald chi‐square test, caste: *χ* ^2^ = 202.614, *df* = 3, *p* < .001, body weight: *χ* ^2^ = 27.346, *df* = 1, *p* < .001). In *Z. nevadensis*, the proportion of polyploid cells in the fat body significantly differed among the castes (GLMM with a Wald chi‐square test, *χ* ^2^ = 71.8927, *df* = 2, *p* < .001), while the effect of body weight was not significant (*χ* ^2^ = 2.706, *df* = 1, *p* = .100). The termite heads comprised a low proportion of polyploid cells (0.034–0.362) and mainly consisted of diploid cells regardless of the caste or sex (Figure 3).

### 3.2 Comparison of the relative polyploid level of queen in the fat body between foraging and wood‐dwelling termites

**Figure 6:**
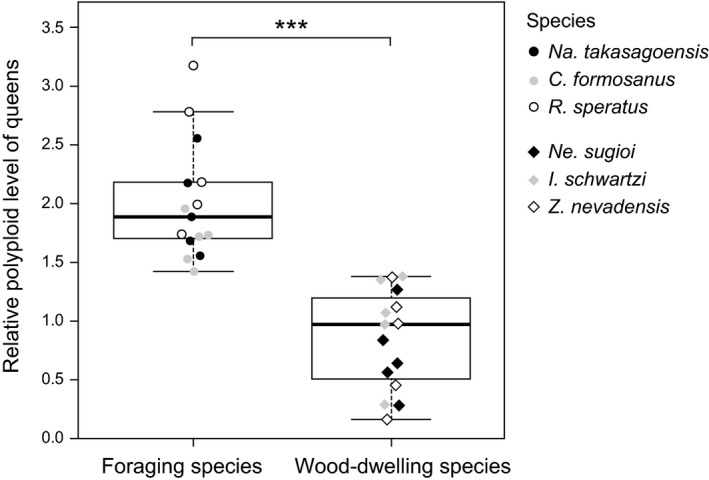
Comparison of relative polyploid levels of queens, calculated based on the conspecific female workers (see text for details). between foraging and wood‐dwelling species. Plots show the median (center line), 75th percentiles (top of box), 25th percentiles (bottom of box), and whiskers connect the largest and smallest values within 1.5 interquartile ranges. Dots are individual values. Asterisk indicates significant differences (linear mixed‐effect model with a Wald chi‐square test, ***p < .001)

We found significant differences in the relative polyploid levels of queen fat body between the species (Figure 6). Queens of foraging species exhibited significantly higher polyploid levels than that exhibited by wood‐dwelling species (LMM with a Wald chi‐square test, *χ* ^2^ = 15.9351, *df* = 1, *p* < .001). On the other hand, differences in body weight between queens and workers did not have significant effect on polyploidy (*χ* ^2^ = 0.012, *df* = 1, *p* = .915).

## 4 DISCUSSION

We found that endoreduplication in the fat body is tightly linked to the reproductive division of labor and the queen fecundity in termites. The fat body of queens had a higher number of polyploid (4C and 8C) cells than that of other nonreproductives in all the six species (Figure 4). In every termite species studied here, castes had significant effects on the rate of polyploid cells in the female fat body, although body weight did not always have a significant effect. These observations suggest that higher proportion of polyploid cells in queen fat body in comparison to that of other females cannot be explained simply by their larger body size. Moreover, males’ fat body, which is independent of vitellogenin synthesis, did not show a consistent pattern of production of polyploid cells based on caste effect or body size; the fat body of kings had more polyploid cells in three of six species but not in other three species (Figure 5). These results do not contradict the idea that the fat body endopolyploidy could contribute to reproductive specialization of queens for egg production. Furthermore, in line with this idea, we demonstrated that in foraging termite species, wherein the queens have high fecundity, the standardized ploidy levels of queen fat body are significantly higher than the levels in the wood‐dwelling species (Figure 6). These findings support the hypothesis that endopolyploidy in the queen fat body is exploited to boost their egg production as an adaptive strategy, whereas the direct and more detailed relationship between egg production and fat body endoreduplication needs to be examined.

Because age‐related endopolyploidy has been reported in worker honeybees (Rangel et al., 2015), it is probably reasonable to expect that the fat body of long‐lived individuals will have more endopolyploid cells than others in termites. In fact, queens in eusocial insects including termites exhibit long lives; their lifespan is more than 10‐fold longer than that of workers and soldiers (Jemielity et al., 2005; Keller, 1998; Thorne, Breisch, & Haverty, 2002). In this study, however, the rate of endopolyploid cells in queen fat body was higher than or equal to that of termite kings, which have extraordinary longevity equal to or greater than the queens (Figures 4 and 5; Boomsma, Baer, & Heinze, 2005). Therefore, age cannot explain the high‐level of endopolyploidy we observed in the termite queen fat body in our study. However, our data cannot exclude the effect of age on the fat body endoreduplication.

Polyploidy plays pivotal roles in the regulation of gene expression, cell size, and differentiation (Edgar et al., 2014; Lee et al., 2010; Neiman et al., 2015). It is possible that the fat body endoreduplication observed in this study is related to the cytological changes as described in previous studies (Costa‐Leonardo et al., 2013; Han & Bordereau, 1982a, 1982b; Šobotník, Weyda, Hanus, Cvačka, & Nebesářová, 2006). Two types of cells are found in the termite fat body: urocytes and adipocytes. Urocytes are unique cells that are packed with urate spherules, while adipocytes are metabolically active cells that synthesize and store lipids, glycogen, and various proteins probably including vitellogenins (Arrese & Soulages, 2010; Costa‐Leonardo et al., 2013). The proportion of these two cell types varies among castes and developmental stages. For example, the fat body of functional queens contains a higher number of adipocytes than urocytes, and the adipocytes of fully matured queens are specialized in the synthesis of proteins; they have limited lipid content but numerous rough endoplasmic reticulum and Golgi apparatus (reviewed in Costa‐Leonardo et al., 2013). Particularly in foraging species, the fat body of queens contains abundant proteins and RNAs, exhibiting considerable differences from that of nonreproductives (royal fat body; Han & Bordereau, 1982a, 1982b). These patterns are similar to our results and, one may reasonably predict that adipocytes show higher ploidy level than urocytes and that the polyploidy can be one of the factors generating the histological modifications in termite queens as mentioned above. A combination of molecular, histological, and cytochemical techniques may help to achieve a better understanding of the relationship of endoreduplication and cellular differentiation in termite fat body.

In this study, using six species that cover the phylogenetic diversity of termites, we successfully demonstrated that the fat body endoreduplication is associated with caste systems and queen fecundity in termites. The six species used in this study belong to four families, Archotermopsidae (*Z. nevadensis*), Kalotermitidae (*Ne. sugioi* and *I. schwartzi*), Rhinotermitidae (*R. spearatus* and *C. formosanus*) and Termitidae (*Na. takasagoensis*). In these taxa, the developmental pathways (linear or bifurcated pathway) and the lifestyle (wood‐dwelling or foraging type) are always coupled, and all foraging species in this study come from a single clade of termites (Bourguignon et al., 2015). Therefore, it may be worthwhile to investigate the ploidy variation in the fat body among a wider range of foraging species, such as harvester termites (*Microhodotermes* and *Hodotermes* species in Hodotemitidae), which are basal species with the bifurcated developmental pathway and highly fecund queens, or the most basal termite, *Mastotermes darwiniensis* Froggatt (Mastotermitidae), in which the queen and workers are developmentally bifurcated, but queens are not fecund unlike the queens in other foraging species (Legendre, Whiting, & Grandcolas, 2013; Myles, 1999). A dry wood termite *Calcaritermes temnocephalus* Silvestri will also provide a unique opportunity to test the hypothesis of endopolyploidy in the fat body and queen fecundity, because queens in the species are highly fecund and exhibit the morphological specialization, which is an exception in Kalotermitidae (Scheffrahn, 2011). The ploidy patterns of these species allow further progress in understanding the evolutionary relationship of extraordinary high fecundity of termite queens and fat body endoreduplication.

In this study, queen types were different among species; queens in *Na. takasagoensis*, *C. formosanus*, *Ne. sugioi*, and *Z. nevadensis* were all adults; however, all queens were neotenics in *R. speratus*. Both neotenic and adult queens were present in *I. schwartzi*. Several developmental and anatomical differences exist between these two queen types (Myles, 1999; Weesner, 1969); nevertheless, in our study, the fat body of all queens exhibited higher levels of endopolyploidy than conspecific nonreproductives, regardless of the queen types. This suggests that the queen types are unlikely to have much of an effect on the ploidy levels in their fat body, although the effect of queen types on endopolyploidy needs to be further examined in a species wherein both adult and neotenic queens are functional.

In conclusion, our study has provided a novel insight into the evolutionary linkage between endoreduplication and caste specialization in social insects. Based on the patterns observed in the study, we suggest the possibility that endopolyploidy in the queen fat body may enhance egg production, mechanisms of which should be assessed in future studies. In future, it will be worthwhile to determine whether polyploid fat body cells show increased expression of genes involved in vitellogenesis. In addition, it will be interesting to determine the cell types of termites that exhibit polyploidy and to explore the proximate mechanisms promoting endoreduplication in the fat body.

## CONFLICT OF INTEREST

The authors declare no conflict of interest.

## AUTHOR CONTRIBUTION

Both authors designed and performed the research. T.N. analyzed the data and wrote the first draft. Both authors then contributed substantially to revisions and approved the final version.

## Data Availability

Data available from the Dryad Digital Repository: https://doi.org/10.5061/dryad.s5v553b.
